# Alveolar Abscess

**Published:** 1880-02

**Authors:** Jno. H. Coyle

**Affiliations:** Thomasvilles, Ga.


### ARTICLE III.

### *Alveolar Abscess.—Origin, Prevention, and Treatment.*

BY JNO. H. COYLE, D. D. S., THOMASVILLE, GA.

There is, perhaps, no subject in the whole range of Dental Pathology which has claimed more careful study than the one which heads this paper; and the result of individual observation, has given to the profession an accurate knowledge of its pathology, and a treatment which, with few exceptions, is specific. Alveolar Abscess is caused by Periodontitis. I prefer this term, because it locates the lesion, and at the same time suggests its pathology which is inflammation of the periosteum lining the alveolus.

The fangs of the teeth are in close contact with highly impressable membrane, and their relationship is so intimate, any disturbance in the one, is followed by a disturbance in the other. It is not necessary for me to enumerate all the causes which may produce this inflammation. The most common cause is from an inflamed or devitalized nerve of a tooth. Whenever the nerve of a tooth becomes devitalized from any cause whatever, and is allowed to remain in the tooth, sooner or later, will produce an alveolar abscess. The dead nerve under this condition, like any other living tissue, when isolated from its source of vitality, is under the dominion of the chemical forces, and disintegration follows. The products of this disintegration are pus and fetid gas. The gaseous product is highly irritating to the periosteum, which it reaches through the special foramen of the tooth. Then the truth of the old medical axiom, „ *Ubiirritatio ibi fluxus*," is fully demonstrated, for this irritation at once invites an increased flow of blood to the seat of the irritation. Then plastic lymph is effused around the inflamed spot, which becomes organized and forms a cyst that catches the product of this inflammation—pus. Then ulceration follows, which gives a free outlet to the confined pus by a fistulous opening through the gum. This is ordinarily the usual course, and result of alveolar abscess, where there is no remedial interference.

As I have stated, this is the exciting cause of the largest number of cases which come to us for treatment, but, we are also called upon to combat periosteal inflammation which is produced by other causes, and if not arrested will result in the death of the nerve and bring about alveolar abscess; so that, under the head of prevention we have to combat Periodontitis. I shall simply give my treatment, remarking that, it is not essential to distinguish the cause of Periodontitis, (unless it proceeds from a devitalized nerve) whether from thermal irritation of the nerve pulp through metal fillings or mechanical violence—the principal is the same, so far as the medical treatment goes. My treatment is local and constitutional. I first paint the gum over the part affected with equal parts of Tinc. Iodine and Tinct. Aconite root as usual, allowing it to dry before allowing moisture to get to it. I then give the patient two powders of saccharated calomel, with directions to take one of the powders at once, and if necessary to take the other powder at an interval of six or eight hours. My experience has been that, it is not often necessary to take but one powder. It is prepared as follows:

| | |
|---|---|
| Calomel, - - - - - - | grs. vi. |
| Powdered White Sugar, - - - - | " xii. |

Rub thoroughly together into mortar and divide in twelve powders.

This looks like Homœpathy, nevertheless, it operates very actively. One of these powders taken on an empty stomach on retiring at night, with most persons is an active purgative. The combination is not original with me. I believe Dr. Batty, of Rome, Ga., first suggested it. The known therapeutical tendency of mercury to affect particularly and prominently the dental tissue, led me to believe that it would be a useful remedy in these cases, and I have found it to be all that could be desired, if its exhibition is not delayed too long. Where it is not desired to use a purgative, I give forty to sixty drops of the Tinct. Gelsem-inum and repeat in one hour if necessary. This often brings needed relief, but is not so certain as the saccharated powders. In addition to this, the exciting cause, if known, must be removed. If it is not, a recurrence of the inflammation may be expected. If it proceed from thermal irritation through metalic fillings, they should be removed and a *non-conducting* substance substituted therefor; and so on through the entire list of causes, whether local or systemic, the proper steps should be taken for their removal. After all efforts fail to control the inflammation, which will sometimes be the case, and the formation of abscess is inevitable, then the only course left is to expose the nerve pulp and devitalize it or extract the tooth. If the first course is decided upon and every part of the devitalized nerve removed, the tooth properly treated and filled, it may be confidently expected that no abscess will occur afterwards. I will now offer some remarks on chronic alveolar abscess. These cases present themselves under one or two conditions namely: a cronic discharge of pus through a fistulous opening from the alveolus or through the cavity of decay. The latter I will designate as closed abscess. The first thing to consider when these cases present themselves is whether it will be advisable to undertake any treatment, other than extraction. All those cases of recent occurrence, on teeth which are sufficiently accessible to admit of the necessary manipulations, may be subjected to treatment looking to their cure, with the exceptions I will presently name. The treatment for abscess with fistulous opening in the gum is very simple and consists in the cure of a pus secreting surface within the alveolus, by the introduction of an Escharotic and antiseptic substance, of the many substances suggested and used for this purpose, I have found nothing superior to carbolic acid full strength, that is, Calvert's carbolic acid with just sufficient water added to make it retain the liquid form. I have never known it to fail where it was properly applied in a case where it was proper to apply any medical substance. If the acid can be forced through the apicial foramen of the tooth, until it appears at the opening of the fistula on the gum, the one application will generally be sufficient. *There should never be any hurry to follow up the treatment by filling;* not less than a week should intervene and *three weeks* will be better. In the filling of these teeth, it should be borne in mind, that *there is no exclusive virtue resident in one material* used for the purpose, but, rather in the *manner* in which it is done. It is absolutely essential that it shall be done thoroughly and with as little violence as possible, making sure that the material used shall fill the entire fang, so that there shall remain no reservoir, to catch a subsequent secretion of pus. A large percentage of abscess managed in this way, are apparently cured and the tooth preserved for future usefulness. This cannot be said to be invariably true, for there is always not only a *possibility*, but a *probability* of a recurrence of the abscess. Those cases which I designate closed abscess. The great difficulty which arises from effort at treating this class of abscess, is the tendency it has to assume the acute form in these cases. When an introduction of the Therapeutic agent is made, and the cavity of decay through which the application is made is closed up for its retention, there is no outlet for the constantly secreted pus within, as is the case when there is a fistula through the gum, and we have a superventive of acute inflammation. Formerly I had a great deal of trouble with these cases, but for the last year I have succeeded in avoiding this trouble by the following treatment; after taking such steps as are necessary to give me free access to the fang canal, I syringe it out with alcohol, by means of an ordinary Hyperdermic syringe, and place a pledget of cotton saturated with the same into it and close up with cotton and sandarach varnish, or after cleansing the cavity as described I place a pledget of cotton saturated with chloroform in the fang canal and close as before, instructing the patient to return in two days, when I introduce carbolic acid, which

I continue to apply, say twice a week until ready for filling. The chloroform treatment *I regard as specific*, as I have never had a case to take on acute inflammation when it was used.

Now, as to the exceptions which should all be treated by extraction—1st. Chronic abscess of long standing. 2nd. When it is evident that a portion of the fang is necrosed· 3d. Abscess of the *Dens Sapientiæ.* 4th. Abscess of the Deciduous Teeth. 5th. All those cases where persons in whom they occur are of scrofulitic diathesis or having cochexia of body by inheritance, predisposing them to malignant growths.

I shall give my reasons for the fifth class of exemptions only, as it seems to me that one, two, three, and four are self evident to all who have had an extensive experience in the treatment of alveolar abscess. All of the Epulic growths of the Maxillary Bones, have their origin in the periosteum of the alveolus. Several cases of tumors of this character have come under my observation within the last ten years and were in my opinion, directly traceable to chronic alveolar abscess of long standing and the patients were of scrofulitic diathesis. All of these cases were benign in character, except one, and radical cure was effected by extracting the tooth and extirpating the alveolus in which the abscess was situated. The case excepted proved to be malignant, and has not yet terminated. The charcter of these Epulic growth, whether benign or malignant, is determined by the peculiar diathesis of the particular individual. Believing, that in these persons, chronic alveolar abscess is often the exciting cause of Epulic growths malignant in character, which may have a fatal termination, I have determined to treat them by extraction only.—*Dental Luminary.*

